# What Shall We Do in the Case?

**Published:** 1889-07

**Authors:** Gilbert E. Corbin

**Affiliations:** St. Johns, Mich.


### What Shall We Do in the Case?

GILBERT E. CORBIN, M.D., D.D.S., ST. JOHNS, MICH.

Read before the Michigan State Dental Society, June 6th, 1889.

Artificial teeth *can* be so inserted as to defy detection. They usually *are* so inserted as to look like signs for crockery stores. Why? Large numbers of persons go "shopping" intent only on procuring their plates at least possible prices, so that manufacturers of such mechanical appliances compete only in *price*, and dwarf or ignore all artistic taste they may possess.

So numerous have these crockery signs become in the community that many patients have actually adopted them as their standard of comparison and positively object to the slightest irregularity of arrangement, to any yellowness of tint, and to many other very close imitations of beautiful natural teeth. Viewed simply as a mechanical trade this is the legitimate result of the high-pressure business methods of the times.

On the other hand, most people of taste, refinement, and culture fully appreciate that artistic ability, in this or any other direction, must be acquired at the expense of years of toil, and can not be purchased at the price of a cheap crockery sign.

Aside from *artistic taste*, correct dentistry demands a *professional* knowledge of health and disease, life and death, the laws governing vitality, physiology, pathology, and kindred sciences. But men possessing this knowledge, if conscientious, will not permit the occasion for many plates of teeth. Knowledge preserves teeth; ignorance destroys them. Imperfect fillings by poor dentists, and neglect by careless patients, soon destroy natural teeth and call for artificial ones. Timely work and perfect work, and proper care on the part of the patient will preserve most teeth for a life time. We all imbibe knowledge through observation from our surroundings. We possess no knowledge whatever on any subject which we have never seen, heard, nor thought of. Patients and others whose knowledge of dentistry has been solely derived from observations of the dentistry of the past, have no just conception of the possibilities in the dentistry of the present. Any firm roots now are far too valuable to be lost, as comfort, usefulness, and durability can be secured by proper treatment and a restoration of their crowns.

By most physicians and by many dentists it was long thought that the extraction of any aching tooth was justifiable and desirable, if not imperatively necessary. With such a standard as that on the part of the profession it became an easy matter for incompetent and unscrupulous parties to persuade patients to pay both for the extraction of good teeth and the insertion of very poor substitutes. Indeed such practices have not been entirely discontinued yet. There is still a great and unjustifiable slaughter of human teeth. Painless extraction, and cheap substitutes, seem to be the prevailing arguments with far too many. Even the street vender of wholesale destruction in this direction is largely patronized.

The credulity that supplies the willing victims not unfrequently excites disgust and contempt; and yet the suffering that comes from ignorance generally appeals very successfully to the sympathy of the public. The great State of Michigan has been very generous in the endowment of charitable institutions. Besides this, yea beyond this, she has enacted wise laws for the protection of the health, the lives, and the limbs of her citizens. She has enacted a law for the regulation of the practice of dentistry, than which there is no more important branch of surgery. The law, though imperfect, has already wrought great benefit. It provides conditions for registration, and a penalty for the practice of dentistry without registration. Notwithstanding all this, street fakirs procure licenses from village boards to conduct the business of indiscriminate extraction of human teeth on the public streets, and on fair grounds.

If it be suggested, "prosecute under the law," I ask who among you will come forward and testify that that is practicing dentistry or any thing akin to dentistry? As well might you define decapitation by the guillotine to be surgery? Indeed the line of demarcation between the practice of dentistry and the mal-practice of dentistry is not as well defined as it should be. I would not take from the general surgeon the privilege of improvising his own splints when called to adjust the fragments of a broken bone. His ability and willingness to do so are to be placed to his credit, and in cases of emergency may be very greatly to the benefit of his patient.

I would not deny to the dental surgeon the privilege of constructing artificial substitutes when needed. I am not willing to concede that such a course is incompatible with his calling, or derogatory to his dignity. We can not concede to the general surgeon the right to fracture bones that he may apply his improvised splints. No one will concede the right to extract valuable teeth for the express purpose of supplying poor substitutes; and yet that very thing is being extensively done all over the country. At any rate, teeth that are susceptible of being made comfortable and serviceable for a long period of years are being extracted in large numbers and their places supplied by cheap, clumsy substitutes.

Why is this so? To whom shall we charge the fault, the patient, or the operator? While a few patients are astonishingly persistent in the wrong direction, it is a fact that most patients accept the advice of the operator unhesitatingly. Are we not then forced to the conclusion that a portion of the public, by far too large a portion of the public, are being erroneously educated in regard to the desirable and possible extent to which human teeth can be saved? From what source is this erroneous education derived? Who are the instructors? Are they physicians? Are they general surgeons? Are they dental surgeons? Are they dentists of any specialty? Are they gentlemen, intelligent and conscientious? By what name shall we know them? The question of registration does not identify them. The matter of diplomas does not sufficiently describe them. They are found on both sides of the line. Will the item of ability throw any light on the question? Are we not inevitably forced to the conclusion that if they could successfully save the natural teeth they would do it?

What then shall we do in the case? From whom and by what means can the public at large derive the much needed information in this matter? Can we accomplish more by any mode of united action than we are doing individually?

The laws of hereditary transmission are now well understood. The offspring is to a large extent stamped with the mental and physical peculiarities of the parents. Intellectual depravity and physical deformity are surely transmissible. The violent destruction of any set of organs, persistently repeated through a series of generations, just as certainly and necessarily results in an impaired and imperfect development of those organs as does daylight follow darkness.

The prospective parent who voluntarily and yet unnecessarily parts with the teeth commits a crime toward his or her yet unborn offspring. Good and perfect natural teeth play so important a part in the animal economy that not even one tooth can be lost without lasting detriment to the whole system. Every organ and every fibre in the body suffer as a consequence. The question of cause and effect in this field has been so thoroughly studied by the most eminent scholars as to well nigh prove the truth of the opinion that on an average human life is shortened one year for each tooth lost. As members of an intelligent and benevolent profession, working not solely for a subsistence, but with a conscientious determination that the world shall be the better for our having lived in it, I repeat the question, Gentlemen, what shall we do in the case?

---

DISCUSSION.

Dr. Dorrance, who opened the discussion, said it was a broad subject and the question suggested itself, What can we do in the case? He thought we could do something by looking at ourselves and seeing that we do not fall into the ways and methods of the so-called crockery manufacturers.

Dr. Long thought there were more good teeth extracted by physicians than by dentists. People in country towns go to their physicians when they want teeth extracted instead of going to the dentist.

Dr. Rix wished to call the attention of the members to the charlatans, with cow-boys and brass bands, who go about the country extracting teeth. One located at Dowagiac for three weeks, and in that time claimed to have extracted 7,000 teeth.

Dr. H. A. Smith, of Cincinnati, was called upon by the chair for remarks on this subject. He said the dental profession is young and becoming more enlightened every year. We expect too much all at once. We must educate our patients to the value of their teeth so they will appreciate and take proper care of them.

Dr. E. C. Moore's paper entitled "Something," consisted in describing and passing around for inspection a number of devices gotten up by himself. They included a small pin vice for holding a separating saw, a jeweler's saw holding a strip of emory cloth, a syringe made from a rubber bulb and German silver tip, a spatula made of German silver for mixing cements, a small enamel chisel, an instrument for keeping corundum points moist while using in the mouth made from a mucilage holder, a pear-shaped mass of lead for holding against the opposite side of a tooth while filling approximal cavities in the anterior teeth with gold, an oil stone mounted on a mandrel for sharpening burrs, and an oil can to contain water for use, in small quantities, in the laboratory.

---

### DISCUSSION OF THE SUPERVISOR OF CLINICS REPORT.

Dr. Moore spoke strongly against the clinic given for extracting teeth and thought it was entirely out of place.

Dr. Harroun was of the same opinion as Dr. Moore and did not see why Dr. Long gave the clinic.

Dr. Parker thought a clinic should be for instruction, not to show the skill of the operator, in future the nature of the operation should be understood beforehand.

Dr. Taft said the clinician should give an explanation of each step in the operation as he goes along so that the crowd would not surround the operator obstructing and embarrassing him in his work.

Dr. Case thought the clinic should be made a feature of dental society meetings. The most progressive societies had the most clinics.

Dr. Dyer said Dr. Taft's clinic at the meeting of the Iowa Society was watched with great interest.

Dr. Wassail, of Chicago, thought clinics were not as useful now as in former days. The coming generation of dentists are mostly dental college graduates and skilled to a certain extent before starting in practice. He thought papers and discussions were becoming of more interest to the profession.

---

Free Medical Advice.—"Doctor," said a citizen, as he overtook him on the street, "what do you do in case of a gone stomach?"

"Well," replied the doctor, thoughtfully, I've never had such a case myself, but I would recommend you to advertise for it, and then sit down in a large easy chair and wait until somebody brings it back."

